# Supplementary material for: Patterns of Intron Gain and Loss in Fungi
Source: PLoS Biol. 2004 Nov 30;2(12):e422. doi: 10.1371/journal.pbio.0020422 (PMC532390; doi:10.1371/journal.pbio.0020422)
Supplement: Table S1 — Also available at http://genes.mit.edu/NielsenEtAl/. (4.3 MB ZIP). [file pbio.0020422.st001.zip › NielsenEtAl/html/285.html]

AN1154.1.NCU08339.1.MG04439.1.FG01013.1


```
 CLUSTAL W (1.82) Multiple Sequence Alignments - Introns Inserted


Sequence 1: MG04439.1	203 aa
Sequence 2: FG01013.1	203 aa
Sequence 3: NCU08339.1	200 aa
Sequence 4: AN1154.1	202 aa
Alignment Length: 203 aa
Number Identitical Residues: 113 aa
Alignment Score (without introns) 5810


MG04439.1 	MLFPSATRLLAAASVLSAALAHNIGLPAHGRECFYENLHKDDKMTVTFQVGDREFGSAGN
NCU08339.1	MQFLTAAKLLLSASLFGAAAAHNIVLPAHGRECFHENLHRDDKMTVTFQVGDREFGSAGN
FG01013.1 	MYFISAAKALVAAAVFSAVTAHNIVLPAHGRECFHESLHKDDTMTVTFQVGDREFGSAGN
AN1154.1  	MRFS-AASLLLGLSWITAAAAHNIQLRAHSRECFHETLHKDDKMTVSFQVGDREFGGSGN
          	* *  *:  * . : : *. **** * **.****:*.**:**.***:*********.:**

MG04439.1 	LDIDFW0ITNPEGQYETFSKSTSNGDYSFTAHLDGQYTYCFGNEHWGATSKEVSFNVHGI
NCU08339.1	LDIDFW0ITNPLNQYETFEKSISNGDFSFEAKHDGKYVYCFGNEHWGANTKEVSFNVHGI
FG01013.1 	LDIDFW0ITNPTGQYETNEKSISNGDYSFTAKHDGKYLYCFGNEAWGANTKEVSFNVHGI
AN1154.1  	LEIDFW0VEDPQSNRQYYKQAISSEDYSFTAQLDGKYVYCFSNEGWTSNSKEVSFNVHGI
          	*:**** : :* .: :  .:: *. *:** *: **:* ***.** * :.:**********

MG04439.1 	VYVSEHDQPQDPLESSV~RQLSELLAQVKDEQSYIILRERTHRNTAESTNSRVKWWNLFV
NCU08339.1	VYISEADAPQDPLEVEV~RKLSEMLEMVKDEQSYIVVRERTHRNTAESTNARVKWWNLFI
FG01013.1 	VYVSEHEMPSDPLDREV1RKLSDLLAQVKDEQEYIVMRERTHRNTAESTNSRVKWWNMFV
AN1154.1  	VYVPESEMAQDPLETEV1RRLLENLAQVKDEQSYIVVRERVHRNTAESTNARVKWWSIFQ
          	**:.* : ..***: .* *:* : *  *****.**::***.*********:*****.:* 

MG04439.1 	ICFIIGEYVFQVWWLRRFFE~VKRVV
NCU08339.1	VGVVVGESLFQVWWLKRFFE~VC---
FG01013.1 	IGVVIGESLFQVWWLRRFFE~VKRVV
AN1154.1  	LAVLIGEGIFQVWWLKRFFE0VKRVV
          	: .::** :******:**** *
```
